# Soil Amendment and Storage Effect the Quality of Winter Melons (*Benincasa hispida* (Thunb) Cogn.) and Their Juice

**DOI:** 10.3390/foods12010209

**Published:** 2023-01-03

**Authors:** Jinhe Bai, Erin N. Rosskopf, Kristen A. Jeffries, Wei Zhao, Anne Plotto

**Affiliations:** USDA, ARS, U.S. Horticultural Research Laboratory, 2001 S. Rock Rd, Fort Pierce, FL 34945, USA

**Keywords:** *Benincasa hispida*, anaerobic soil disinfestation, ASD, volatile, soluble solids content, brix, acid, zeta potential, particle size, amino acid, vitamin, nucleoside, long storage

## Abstract

Winter melon fruits were grown in the field using anaerobic soil disinfestation (ASD) and conventional fertilizer alone as the control treatment. Fruits were harvested and stored at 20 °C for 120 d, the juice was processed on day one and day 120, and the effects of soil amendment and 120 d storage on the juice’s physical and chemical (sugars, acids, volatile and nutritional compounds) properties were evaluated. Fruit juice extracted from ASD-grown fruit had greater magnitude of zeta potential than the control juice, indicating it was physically more stable than the juice obtained from the control conditions. ASD fruit juice had lower soluble solids content (SSC), and lower volatile compounds that contribute green, grass, and sulfur notes, and negatively influence flavor quality. ASD fruit juice had higher vitamin B5 and cytidine. Juice processed from 120 d stored fruit had less yield due to 12.4–15.6% weight loss. The non-soluble solids content was higher and particle size was larger, and the SSC and individual sugars decreased. However, titratable acidity (TA) increased primarily due to increased citric acid. Out of 16 free amino acids, 6 increased and only 1 decreased. However, three out of five nucleosides decreased; vitamins B1 and B6 increased; vitamins B2, B3 and C decreased. Overall, juice derived from fruit produced using ASD was physically more stable and had less SSC and off-odor volatiles than the control, while the fruit juice of those stored for 120 d had lower SSC and higher TA and nutritional profiles, comparable to freshly harvested fruit.

## 1. Introduction

There is sufficient scientific research and surveys showing that decreasing sugar-sweetened beverage (SSB) intake will reduce the prevalence of overweight individuals and obesity [1,2,3,4]. In contrast, increased fruit and vegetable intake lowers the risk of serious health problems, such as cardiovascular diseases and cancer [5,6,7]. Some fruit juice has similar energy density and sugar content to SSBs, and high fruit juice intake can be associated with an increased risk of diabetes in spite of the fact that fruit juices also contain vitamins, fibers, and minerals [8,9,10]. Thus, the search for low-sugar juices and drinks is motivated by public health concerns [11].

Different from the sweet cucurbits, such as melons [12] and watermelon [13], winter melon (*Benincasa hispida* (Thunb) Cogn.) is a non-sweet cucurbit (<3% sugars) with >90% of water content [14,15]. It is grown in temperate to tropical regions worldwide [14] and the crop grows well in Florida [16]. Winter melon is often recognized for its nutritional and medicinal properties, especially in Asian countries [14,17]. It is a good source of amino acids (such as three phenolic amino acids [18]), organic acids (tartronic acid with great potential to play a role in inhibiting the conversion of carbohydrates into fats [19,20]), mineral elements (such as Ca, K, Mg, Fe, Se [14,21]) and vitamins (such as vitamin C, riboflavin and Niacin [14]). A number of medicinal properties such as anxiolytic, anticonvulsant, antidepressant, anti-inflammatory and analgesic, antiasthmatic, hypolipidemic effects have been observed from winter melon fruit [14,17,21,22]. Pharmacological activities have also been revealed in seeds, fruit peel and plant stems [22]. 

Previous research showed that winter melon juice contains less than 3% soluble solids content (SSC), and the juice has potential for stand-alone and blends with other fruit and vegetable juices [18,23]. For this study, we compared different winter melon cultivars and selected a waxy-coated, round cultivar that produces low sugar, with physically stable juice (less cloudloss) and less off-odor [18].

Anaerobic soil disinfestation (ASD) is a method of soil disinfestation that is under development for use in crops that were previously produced using the fumigant methyl bromide prior to planting. In Florida, ASD is based on the incorporation of a labile carbon source combined with pasteurized poultry litter, irrigation to soil saturation, and tarping with gas-impermeable film for about three weeks [24]. The growth of soil anaerobic microorganisms increases as a direct response to a readily available carbon source and anaerobic conditions, producing organic acids as a result of anaerobic decomposition. These compounds are often lethal to soil-borne plant pathogens and plant-parasitic nematodes. Moreover, changes in resident soil microbial communities, fostered by resource availability in soil and the rhizosphere, may actively contribute to disease suppression. While it was reported that ASD affected plant growth and increased tomato yield [25], there are no published reports on the effects of ASD on fruit quality of winter melons.

In Florida, winter melon harvest season starts in late May/early June and ends in late January/early February [16]. To secure a stable supply of fruit for processors, technology needs to be developed to provide fruit during the four-month off-season. Typically, fruit are stored in cold temperatures for extended periods of time. However, similarly to other chilling sensitive fruit, such as mango [26] and Japanese apricot [27], winter melons suffer chilling injury when stored below 13 °C [15,28,29]; therefore room temperature is recommended for long-term storage [15]. A long-term storage experiment showed that many winter melon breeding lines could be stored at ambient temperature up to 120 d [30]. During storage, sugar, crude fiber, calcium and dry matter content increased while ascorbic acid and pectin content decreased [30]. However, to date, there is limited knowledge of how fruit quality and its juice are affected by long-term storage.

The objectives of the current research were to determine (1) if soil amendment by ASD affects winter melon fruit storability and juice processing properties and (2) the effects of long-term storage of the fruit on juice quality.

## 2. Materials and Methods

Winter melon [*Benincasa hispida* (Thunb.) Cogn] cv. ‘Large Round’ (round type with thick waxy coating) was directly seeded into raised beds treated with either ASD or fertilizer only, at the USDA Horticultural Research Laboratory Picos Farm in Fort Pierce, Florida, USA in fall 2020. After 120 d, fruit were harvested on 3 February 2021 and stored in a 20 °C dark room for up to 120 d. Fruit weight was measured during storage, and juice was extracted from 1-day and 120-day stored fruit. Juice processing properties and physicochemical attributes were determined, and effects of soil amendment and storage time on fruit and juice quality were compared. The experiment was designed with three field replicates. 

### 2.1. Plant Materials and Field Treatment

Six raised beds (30 m long, 0.9 m wide, 0.25 m high and 1.8 m between centers) were constructed and three each randomly assigned to ASD or control to represent three field replications. Each control bed received a commonly implemented application of pre-plant compound fertilizer (10N-10P_2_O_5_-10K_2_O) at a rate of 560 kg ha^−1^; supplemental liquid fertilizer was applied via irrigation as needed. Pasteurized, pelleted poultry litter (ChickMagic, Cold Spring Egg Farm, Inc., Whitewater, WI, USA) applied at 15 mg ha^−1^ and feed-grade, black strap molasses (Double S Liquid Feed Service, Danville, IL, USA) at 13.9 m^3^ ha^−1^ was used to establish ASD treatments [31]. Amendments were applied to the bed top and incorporated to a soil depth of 15 cm using a rotary cultivator. VaporSafe^®^ totally impermeable film (TIF, 0.03 mm white on black, Raven Industries Inc., Sioux Falls, SD, USA) was used to cover all the beds. Each ASD-treated bed was irrigated with 5 cm of water applied through two drip lines established on each bed [32]. Winter melon was direct-seeded four weeks after ASD treatments were initiated using in-bed spacing of 1.22 m between holes and three seeds per hole. Plants were thinned to two plants per hole after emergence. Each replicate contained 50 plants. A single harvest of fruit sized larger than 4.54 kg was conducted 120 d after planting. 

### 2.2. Fruit Storage and Weight Loss 

**Figure 1:**
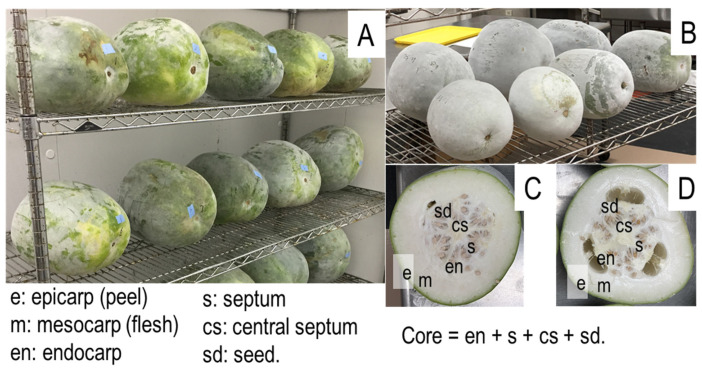
Surface waxy coat and internal structure of winter melon fruit at harvest and after 120 d storage at 20 °C. (**A**): Fruit, at harvest, covered with different levels of waxy coating; (**B**): Fruit, after 120 d storage at 20 °C, fully covered with thick waxy coating; (**C**,**D**): Transverse section of fruit at harvest (**C**) and after 120 d storage at 20 °C (**D**).

Melons were held on trellis shelves without packaging, and space between melons was maintained in storage (Figure 1A,B). Storage temperature was 20 °C, with relative humidity of 80%. After one or 120 d storage, three melons × three replicates per treatment were processed. For weight loss measurement, fruit weight was measured weekly. 

### 2.3. Juice Processing 

Juice samples were extracted from melons in a lab-scale processing system. Briefly, after careful washing with fruit detergent (Fruit Cleaner 395, JBT Food Tech., Lakeland, FL, USA), melons were rinsed, and air-dried. After removing the peel (about 3 mm) and core (Figure 1C,D), the flesh was cut into 2 cm cubes, and juiced using a Juicerator (Model 6001, Acme Juicer Mfg Co, Sierra Madre, CA, USA) with a milk filter (Schwartz Manufacturing Co., Two Rivers, WI, USA) at 2500–3000× *g* for 1 min. Then, the juice was centrifuged at 17,000× *g* for 30 min, and pulp-free juice samples were collected for further quality analysis. Pulp from the filter and centrifuged pellets were merged as “pulp”. Aliquots of the supernatant were taken for dry juice weight, soluble solids content (SSC), titratable acidity (TA), volatiles and non-volatiles analysis, and the measurements were taken from freshly harvested fruit or after storage at −80 °C (volatiles) for up to 4 months.

### 2.4. Juice Yield and Processing Waste

Material weight was taken at each processing stage, including whole fruit after wash, peel, flesh and core, filtered pulp and centrifuged pellet (pulp) and pulp-free juice. Thus, juice production, edible portion (flesh), and waste were calculated.

### 2.5. Physicochemical Quality Attributes

#### 2.5.1. Particle Size and Juice Stability 

In order to study the particle size, zeta potential, and juice stability, juice samples, 15 mL × 3 test tubes per replicate, were heated at 100 °C in a water bath for 10 min to inactivate the enzymes. After cooling the juice samples to 25 °C under tap water, 45 mL juice sample per replicate was collected and centrifuged for 10 min at 360× *g*. The supernatant was used for particle size, zeta potential, and juice opacity measurements. For particle size and zeta potential, the samples were analyzed using a Zetasizer (Malvern Panalytical, Malvern, UK). For juice opacity, juice samples were transferred to a cuvette and absorbance at 660 nm was recorded using a UV–visible spectrophotometer (UV-2401PC, Shimadzu, Columbia, MD, USA) [33]. High absorbance readings correspond to high opacity.

#### 2.5.2. Juice Dry Matter and Non-Soluble Dry Matter

Juice, 100 g per replicate, was vacuum-dried at 55 °C. The total dry matter was directly measured, and non-soluble dry matter was obtained by subtracting SSC from the total dry matter. All were expressed as percentage of total juice. 

#### 2.5.3. SSC, pH and TA

Juice SSC was measured from the supernatant with a refractometer (RX5000α, Atago, Tokyo, Japan). TA and pH were measured by using a titrator equipped with a robotic autosampler (model 855, Metrohm, Herisau, Switzerland), a dosing interface (Dosino model 800, Metrohm, Herisau, Switzerland) and controlling software (Tiamo v. 2.5, Metrohm, Herisau, Switzerland). The principle procedure for TA determination was to titrate 10 mL of juice sample with 0.1 N NaOH to pH 8.1, the TA value was expressed as [H^+^] concentration, mmol L^−1^ [34].

#### 2.5.4. Measurement of Volatile Compounds

Juice sample, 6 mL, was transferred to a 20-mL vial sealed with Teflon-lined septa (Gerstel Inc., Linthicum, MD, USA). Volatiles were analyzed by headspace (HS)—solid phase microextraction (SPME)—gas chromatography (GC)—mass spectrometry (MS) system, equipped with an autosampler and a cooling system to maintain samples at 4 °C [35,36]. Juice samples were pre-heated for 30 min at 40 °C. A 2 cm SPME fiber (50/30 µm DVB/Carboxen/PDMS, Supelco, Bellefonte, PA, USA) was then exposed to the headspace for 60 min at 40 °C. After exposure, the SPME fiber was inserted into the GC-MS (Model 7890 GC and 5975 N MS, Agilent, Santa Clara, CA, USA) injector to desorb the extract for 15 min at 250 °C. A DB-5 column (60 m × 0.25 mm i.d., 1.00 µm film thickness, J&W Scientific, Folsom, CA, USA) was used to separate volatiles. MS settings were from 30 to 250 *m/z* and ionized at 70 eV. Volatile compounds were identified by matching their spectra with those from the National Institute of Standards and Technology (NIST)/Environmental Protection Agency (EPA)/National Institutes of Health (NIH) Mass Spectral Library (NIST 14; WebBook, SRD69) and authentic volatile compound standards, as well as by comparing their RIs with corresponding literature data [23]. The concentrations of volatiles in the juices were calculated from the total ion current (TIC) of headspace samples, using the regression equations, determined by injecting five sequential concentrations of each standard to obtain a TIC calibration [37]. The calibration curves were constructed by adding chemical standards (Sigma-Aldrich, St. Louis, MO, USA) to a deodorized juice matrix. Deodorization of juice was performed by a rotary evaporator (R-215, Büchi, Flawil, Switzerland) equipped with a heating bath (B-491) set at 40 °C, a recirculating chiller (F-114) set at −3 °C, and a vacuum controller (V-855) operated under 70–20 mbar with a rotary speed 200–240 rpm until SSC reached 45%. Deodorized juice was then reconstituted to SSC 2.14% (original SSC level) by using DI water as the deodorized juice matrix. The chemical standards with C6 or smaller molecules were added directly into the deodorized juice matrix. The chemicals with >C6 molecules were prepared in two steps, first adding the standards into methanol to make a 1% stock solution; then, the stock solutions were added to the deodorized juice matrix. All chemicals were mixed, and the concentrations were based on the abundance of each chemical in the juice samples. The concentration ranges were 31.25–500 µL L^−1^ in hexanal, (*E*)-2-hexenal and 1-hexanol, 0.3125–5 µL L^−1^ in acetaldehyde, methanethiol, dimethyl sulfide, pentanal, and dimethyl disulfide, and 1.5625–25 µL L^−1^ in all others. Each mixture had three replicates that were averaged. 

#### 2.5.5. Measurement of Nonvolatile Compounds

Juice (supernatant) was filtered with a 0.2 µm PES syringe and spiked with an internal standard, ^13^C_6_-D-fructose, at a final concentration of 40 mg L^−1^. LC-MS/MS analyses were performed with an 1290 Infinity II UPLC coupled with a 6470 triple quadrupole MS (Agilent, Santa Clara, CA, USA). The InfinityLab Poroshell 120 HILIC-Z column (2.1 × 150 mm, 2.7 µm, Agilent, Santa Clara, CA, USA) was held at 15 °C. Mobile A consisted of water, 20 mM ammonium acetate (pH 9.3), 5 μM medronic acid and mobile B was acetonitrile. Initial UPLC conditions were 90% B, 10% A for 1 min, followed by a linear gradient to 78% B at 8 min, 60% B at 12 min, and 10% B at 15 min. After flushing with 10% B for 3 min, the column was re-equilibrated with initial conditions (90% B, 10% A) for 10 min. The flow rate was kept constant at 0.4 mL min^−1^. The Agilent Jet Stream ESI source was operated with a gas temperature of 225 °C, gas flow of 9 L min^−1^, nebulizer pressure of 30 psi, sheath gas temperature of 375 °C, sheath gas flow of 12 L min^−1^, and a capillary voltage (+ and −) of 3000 V. The MS was operated in dMRM mode and compound-specific fragmentor and collision energy voltages are listed in Supplementary Table S1. Compounds were identified by the comparison of MRM transitions listed in Supplementary Table S1 and retention times with analytical standards. Quantification of the identified compounds was performed by integrating the area under the chromatographic peak and calculating the amount of each compound based on standard curves. Standard curves were run in groups according to abundance levels [amino acids (1–90 mg L^−1^); higher abundant sugars (150–12,500 mg L^−1^); lower abundant sugars and organic acids (10–250 mg L^−1^); nucleosides (0.004–6 mg L^−1^); vitamins B2 and B7 (0.02–1 mg L^−1^); vitamins B1, B3, B5, B6 and B12 (0.03–1 mg L^−1^); vitamin C and malic acid (90–5000 mg L^−1^)]. A minimum of five points were used in each standard curve (R^2^ ≥ 0.99). The vitamin C and malic acid standards were prepared fresh immediately before their injection while the other standards were stored as stock solutions and then diluted with LC/MS water before injection. Each treatment group had three replicates that were averaged. MassHunter Quantitative Analysis software (Agilent, Santa Clara, CA, USA) was used for data analysis.

### 2.6. Statistical Analysis

All measurements were replicated three times using the three field replications. JMP Version 16 (SAS Institute, Gary, NC, USA) was used for analysis of data. Analysis of variance (ANOVA), and principal component analysis (PCA) was used to evaluate the effect of soil treatment and storage time on juice processing and quality attributes. Two independent samples *t*-Test (TTEST) was used to compare the effect of soil and storage treatments.

## 3. Results and Discussion

### 3.1. Weight Loss during Fruit Storage 

**Figure 2:**
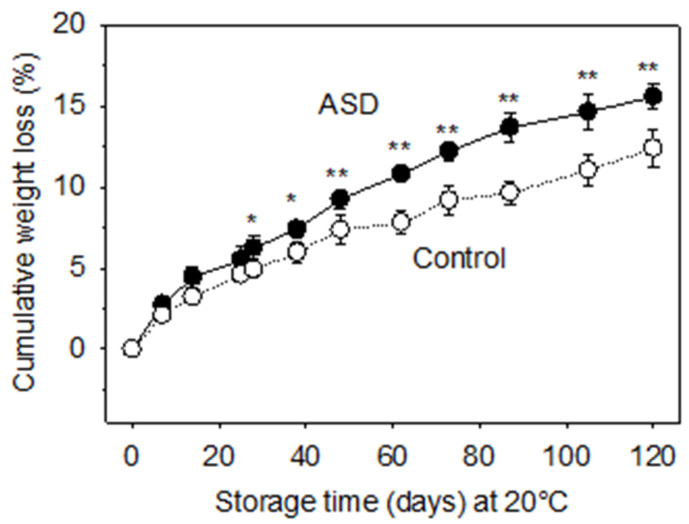
Comparison of weight loss of fruit grown in ASD (black dots) and non-treated soils (white dots). Fruit was stored at 20 °C for up to 120 d. * or ** indicate significant difference at 0.05 or 0.01, respectively, between ASD and control at each storage time.

Fruit harvested from the ASD treatment lost 15.6% of the total weight after 120 d storage, 3.2% more than the control fruit (Figure 2). The greater weight loss in ASD fruit was observed from the beginning and throughout the entire storage time (Figure 2). In the first week, the daily weight loss in ASD fruit was 0.39%, decreased to 0.24% in the next three weeks, further decreased to 0.10–0.18% until 87 d storage and finally decreased to 0.05–0.06% daily (Figure 2). The control fruit had a 0.30% daily weight loss in the first week, decreased to 0.10–0.16% in the following seven weeks, and further decreased to 0.03–0.09% in the last ten weeks (Figure 2). The higher weight loss rate for ASD fruit could be attributed to the lower maturity of the fruit at harvest as observed by a reduced waxy coating cover (Figure 1). Nevertheless, the difference in daily weight loss between the two soil treatments remained even after 60 d storage after the waxy coating had fully developed on both ASD and control fruit (Figure 1).

The transversal section of fresh fruit showed that the cavity was full of endocarp tissue, septum, central septum and seeds at harvest, but after 120 d storage, the endocarp tissue shrank (Figure 1C,D). However, soil treatment and storage did not significantly affect the total core tissue weight (Table 1).

Water loss leads to softening, shriveling, discoloration, and general deterioration of appearance for most fruits and vegetables. Generally, when weight loss exceeds 4 to 6% of total fresh weight, the fresh produce becomes unmarketable or unappetizing [38,39]. However, we did not observe visible deterioration on the surface or in the flesh of fruit after 120 d storage even when the cumulative weight loss was over 15.6%. Similar results were reported in pumpkins that lost 18–21% weight during 120 d storage at 27–31 °C without visible deterioration [40].

### 3.2. Juice Production and Waste

The control and ASD-produced fresh fruit had 67% and 65% flesh (mesocarp, Figure 1C,D), respectively, and changed little during the 120 d storage (Table 1). Juice production was 11.33% and 10.36% lower by weight than the flesh in control and ASD fresh fruit, respectively, due to the pulp being removed by filtration and centrifugation of pellets (Table 1). The pulp had more than 90% water content (data not shown) and could possibly be reduced by increasing filtration pressure; pulp waste could also potentially be used as a source of fiber-rich food supplement. The winter melon juice yield was between 53.70–56.68% of total fruit weight regardless of the soil treatments or storage time, much higher than orange juice yield, which is lower than 50% of total fruit weight [41]. ASD fruit produced more juice with less pulp (Table 1). During the long storage period at 20 °C, fruit maturation progressed, resulting in more pulp (Table 1).

### 3.3. Particle Size and Stability of Juice

Cloud stability is a desirable attribute in fruit juices; it depends on the surface charge of particles in the juice (charge repulsion can keep the cloud particles from aggregating) and the particle size. The zeta potential is a key indicator of the stability of particles in juice, which characterizes the charge of the particle surfaces that affect the precipitation of particles in juice. The higher the absolute value of the zeta potential (negative or positive), the less likely the particles in a cloudy juice will adhere together to form aggregates (flocculation) and sediment, resulting in more stable the juice. Zeta potential and particle size analyses have been proven to be valuable tools for prediction of juice stability and shelf life [42,43,44].

At the same storage time, particle size in ASD juice was smaller than in control juice; and the absolute values of zeta potential of ASD juice samples were larger than those of the control juice samples (Table 2), indicating ASD juice was more stable than control juice. However, based on the criterion that particles with an absolute value of zeta-potential larger than 15 mV are expected to be stable [45], both the ASD and the control juice could not be considered as “stable”, because their absolute values of zeta potential were less than 15 mV, which were 11.58–11.68 mV and 8.77 mV, respectively, for ASD and control juice. Storing fruit for 120 d did not affect the zeta potential value of the juice, though storage increased the particle size in both ASD and control fruit juice (Table 2). Taking the particle size and zeta potential data together, the juice from fresh ASD-grown fruit was more stable than that derived from the control fruit.

### 3.4. Sugars, Acids and Non-Soluble Dry Matter of Juice

The SSC of fresh ASD fruit was 2.14 °Bx, and remained during 120 day storage, however, the value was 2.43 °Bx in the control juice, significantly higher than the ASD fruit juice (Table 3). Interestingly, SSC in the control juice decreased during the 120 d storage period, in spite of the 13–16% fruit water loss (Figure 2 and Table 3). Our results do not agree with the Pandey et al. [30] report, which showed that the SSC increased in all 19 genotypes of winter melons stored at room temperature. In many fruits, the increase in SSC is usually due to the hydrolysis of starch into soluble sugars or as a consequence of water loss [46,47]. The cultivar used in this experiment underwent a decrease in soluble solids likely due to increased respiration and/or metabolic reactions that convert soluble solids to insoluble, as has been reported for many other fruits and vegetables [47]. Fructose and glucose, which account for about 40–50% of SSC in fresh juice, decreased to approximately 35–40% after 120 d storage (Table 3). Wills et al. [48] reported that glucose and fructose in mature winter melon fruit were 0.5% each—similar to our results (Table 3), however, the levels were as high as 0.8–0.9% in immature fruit, indicating that mature fruit should be used if juice with low sugar content is desired. Arabinose, galactose, and xylose, the non-cellulosic neutral sugar components of cell walls were detected, and arabinose was the predominant component (approx. 0.1%), followed by galactose (approx. 0.015%) and xylose (approx. 0.0005%) (Table 3). The results are different from Gross and Sams [49] who reported that galactose was the major component in cucurbit fruit.

Winter melon juice was slightly acidic, with a pH value of 5.5 when fruit was fresh, and increased in acidity to pH 5.2 after 120 d storage (Table 3). The TA was 7.71 and 6.09 mmol L^−1^ in fresh ASD and control juice samples, respectively, and almost doubled after 120 d storage (Table 3). However, malic acid, the most abundant acid, decreased approximately 30% after 120 d storage. Conversely, citric acid increased 5–11 times during storage, and became almost comparable to malic acid; thus the sum of malic and citric acids increased by 10–15% following storage (Table 3).

Tartronic acid occurs naturally in cucumber and winter melon, and may play a critical role in inhibiting the conversion of carbohydrates into fats in animal studies [19,20]. Cucumber and winter melon breeders often set tartronic acid content as a quality attribute [50]. However, our data showed that tartronic acid contents were about 1% of the total acids, and storage did not significantly alter this content (Table 3).

The non-soluble dry matter, an attribute closely correlated to the dietary fiber content [18], was significantly higher in the control juice, and the content significantly increased during storage (Table 3), indicating that along with maturity progress and water loss during storage, cell wall cellulose, hemicelluloses and lignin accumulated (Table 3). Similar results were found in many plants [51], and confirmed with the storage of winter squash [52].

### 3.5. Nutritional Composition of Juice

Six vitamins, 17 free amino acids and five nucleosides were detected by LC-MS/MS (Table 4). Vitamin C content in fresh juice was 135–140 mg L^−1^, which agrees with a previous report in which the extraction solvent was 3% metaphosphoric acid [53]. After 120 d storage at 20 °C, vitamin C was reduced by 25% (Table 4). Five B vitamins were detected: vitamin B5, B1, B6, B2 and B3 (Table 4). The abundant vitamin B5 was higher in ASD fruit samples (Table 4). After 120 d storage, vitamins B1 and B6 increased, but B2 and B3 decreased (Table 4). B7 was not detected in winter melon juice.

Glutamine was the most abundant amino acid, followed by arginine, tryptophan, aspartate, tyrosine, phenylalanine, isoleucine, and valine (Table 4). The total amino acids were 604 and 794 mg L^−1^ at day 1 and increased to 900 and 899 mg L^−1^ after 120 d storage, in ASD and control fruit juice samples, respectively, with no significant differences between the two soil treatments (Table 4). The data agreed with previous reports: tryptophan, tyrosine, phenylalanine, the three phenolic amino acids were major components [18,23]. Dong et al. [54] reported similar total amino acids in winter melon pulp, although the major components were different [14,54].

Nucleosides and nucleotides are essential in a large number of biological processes. They are the precursors of the nucleic acids that make up DNA and RNA. Moreover, nucleosides and nucleotides participate in other metabolic functions as they form part of biosynthetic routes, operate in the transfer of chemical energy, are components of some co-enzymes, and play an important role as biological regulators [55]. Five nucleosides were detected, with adenosine as the greatest component, followed by guanosine (Table 4). Adenosine, inosine and uridine decreased after 120 d storage, and cytidine and total nucleosides were higher in the ASD-derived fruit juice samples than in the control (Table 4).

**Figure 3:**
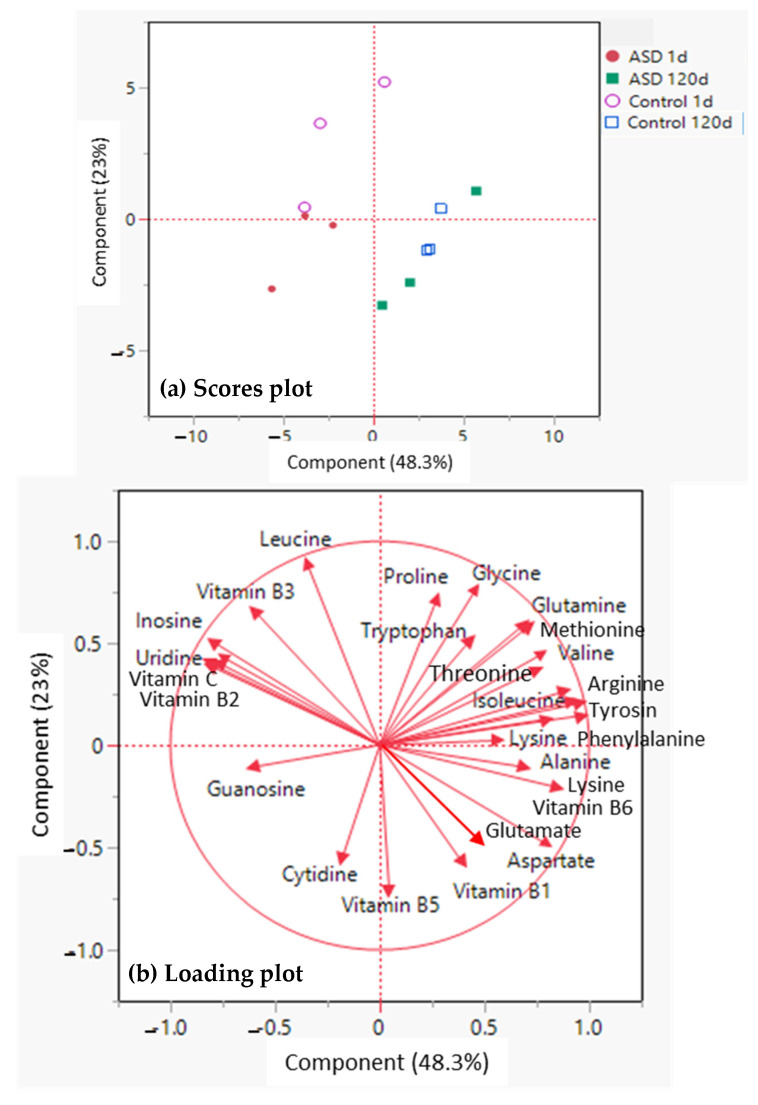
Principal components analysis (PCA) of 6 vitamins, 17 amino acids, and 5 nucleosides in winter melon juice samples extracted from ASD treated or control fruit that were stored at 20 °C for 1 or 120 d. (**a**) Scores plot and (**b**) Loading plot.

A PCA was performed to project the juice nutritional components onto a 2-component plot (Figure 3). The PCA discriminated 120 d storage fruit samples from 1 d storage on both Component 1 and Component 2, explaining 71.3% of the variation in the first two components (Figure 3a,b). Nutritional components from fresh fruit were on the upper-left side, including vitamin C, B2 and B3, leucine, and all nucleosides (guanosine and cytidine being on the lower left part of the quadrant) (Figure 3b). However, most of the amino acids, located on the right side (Figure 3b), were associated with 120 d stored samples.

### 3.6. Volatile and Non-Volatile Profile of Juice

Fifteen volatile compounds were identified by GC-MS (Table 5). The major compounds were aldehydes, including acetaldehyde, pentanal, (*Z*)-3-hexenal, hexanal, (*E*)-2-hexenal, (*E*,*E*)-2,4-hexadienal, nonanal, (*E*,*Z*)-2,6-nonadienal, and (*E*)-2-nonenal. They accounted for about 97% of the total volatiles as measured by total ion current (Table 5). The key flavor notes associated with these volatiles include green, grass, fat and vegetable soup, descriptors which are not favorable in stand-alone juice or juice blends [56,57]. There were three sulfur compounds, methanethiol, dimethyl sulfide, and dimethyl disulfide, which were very low in concentration, but could potentially contribute strong off-odor [58,59]. There was one monoterpene hydrocarbon (d-limonene) with a very light citrus note, one alcohol (1-hexanol) with green/grass note, and a rare compound, methoxy-phenyl oxime which has been reported sporadically without a clear flavor descriptor [60].

**Figure 4:**
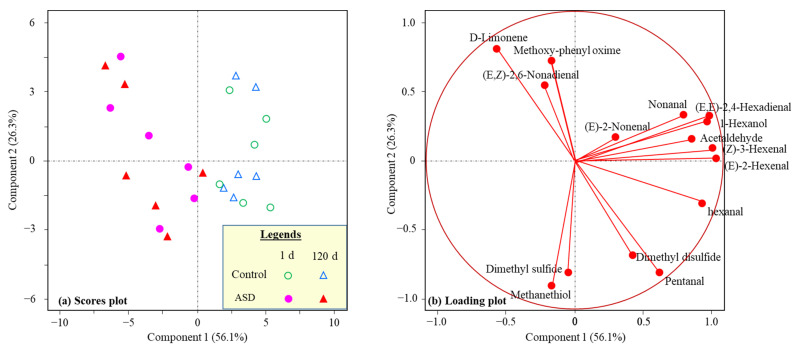
Principal component analysis (PCA) of 16 volatile compounds in winter melon juice samples extracted from ASD treated or control fruit that were stored at 20 °C for 1 or 120 d. (**a**) Scores plot and (**b**) Loading plot.

Most compounds were affected by soil treatment, with the ASD fruit having lower volatile concentration in 11 of 15 compounds, and the total concentration was 25% lower than in the control fruit (Table 5). Those volatiles generally contribute to “green/vegetable” notes, which is not desirable for fruit juice; thus, ASD fruit and juice are presumed to have better flavor quality. Lower volatile abundance is preferred regardless of whether the winter melon juice is used for stand-alone or blended fruit juice. Volatiles were not affected by storage duration, and there was no interaction between soil treatment and storage times (Table 5). PCA analysis confirmed the conclusion that volatile profiles were discriminated by ASD treatment, but not storage time (Figure 4). The PCA discriminated ASD-derived fruit juice samples from the control samples on Component 1, which explained 56.1% of the variation (Figure 4a,b). Most juice volatiles were associated with the control samples, indicating more off-odor volatiles in the control juice (Figure 4).

From this work, we conclude that the gap in fresh fruit supply during the four-month off season in winter melon could be made up by long-term, room temperature stored fruit. The long-term storage resulted in substantial water loss, but there was no loss due to decay. The long storage also did not cause consistent loss of flavor or nutritional quality. This research provides strong evidence to the industry that low-cost room temperature storage would ensure stable fruit supplies for the winter melon juice industry.

## 4. Conclusions

Fruit harvested from the ASD soil amendment treatment had higher juice yield due to reduced core and peel waste. However, ASD fruit lost more weight during 120 d storage at room temperature. Juice from ASD treated fruit was more stable with a greater magnitude of zeta potential, and had lower SSC, non-soluble dry matter, and total volatiles. The volatile components were primarily sulfur compounds, and carbon-6 aldehydes, principally associated with off-flavor in juice. Juice samples resulting from ASD-produced fruit had higher vitamin B5 and cytidine in their nutritional profiles. Storing fruit at 20 °C for 120 d caused fruit weight loss of 12.4 to 15.6%. However, storage did not cause significant changes in the volatile profiles, although it increased juice pulp, non-soluble dry matter, and dramatically increased TA content, which was due primarily to an increase in citric acid. Storage also decreased SSC and pH. Six out of 16 free amino acids increased and only one decreased; however, three out of five nucleosides decreased. Vitamins B1 and B6 increased, but vitamins B2, B3, and C decreased. The results clearly support the conclusion that 120 d storage at 20 °C did not cause an increase in SSC or flavor deterioration, and the juice’s nutritional quality was comparable to that obtained from fresh fruit. Fruit juice from the ASD treatment was physically more stable.

From this work, we conclude that the gap in fresh fruit supply during the four-month off season in winter melon could be made up by long-term, room temperature stored fruit. The long-term storage resulted in substantial water loss, but there was no loss due to decay. The long storage also did not cause consistent loss of flavor or nutritional quality. This research provides strong evidence to the industry that low-cost room temperature storage would ensure stable fruit supplies for the winter melon juice industry.

## Figures and Tables

**Table 1:** Effect of soil treatment and storage time on juice production and waste of winter melons.

Storage Time (d)	Percentage of Each Fruit Portion (%)				ANOVA (Prob > |t|)		
	1		120		Soil Treatment (S)	Storage Time (T)	S × T
Soil Treatment	ASD	Control	ASD	Control			
Flesh	67.05	65.02	67.58	66.84	0.033 * ^z^	0.060	0.263
Juice	56.68	53.70	55.43	54.62	0.008 **	0.772	0.081
Peel	11.48	11.69	12.01	12.36	0.693	0.401	0.922
Core	21.47	23.29	20.41	20.80	0.309	0.118	0.503
Pulp	10.36	11.33	12.15	12.22	0.170	0.005 **	0.231
Total waste	43.32	46.30	44.57	45.38	0.009 **	0.741	0.081

^z^ *, ** represent significant levels at 0.05, and 0.01, respectively.

**Table 2:** Effect of soil treatment and storage time on particle size and juice stability of winter melon juice samples.

Storage Time (d)	Percentage of Each Fruit Portion (%)				ANOVA (Prob > |t|)		
	1		120		Soil Treatment (S)	Storage Time (T)	S × T
Soil Treatment	ASD	Control	ASD	Control			
Absorbance of supernatant (OD 660 nm)	0.122	0.063	0.214	0.154	0.0001 *** ^z^	0.0048 **	0.0064 **
Particle size (nm)	533.1	682.4	700.4	748.2	0.0001 ***	0.0001 ***	0.0215 *
Zeta potential (mV)	−11.580	−8.769	−11.680	−8.767	0.0008 ***	0.2614	0.1843

^z^ *, **, *** represent significant levels at 0.05, 0.01 and 0.001, respectively.

**Table 3:** Effect of soil treatment and storage time on total dry matter, soluble solids content, pH, titratable acidity, non-soluble dry matter and individual sugars and acids of winter melon juice samples.

Storage Time (d)	Percentage of Each Fruit Portion (%)				ANOVA (Prob > |t|)		
	1		120		Soil Treatment (S)	Storage Time (T)	S × T
Soil Treatment	ASD	Control	ASD	Control			
Total dry matter (%)	2.23	2.57	2.25	2.42	0.0010 *** ^z^	0.1229	0.0630
Soluble solids content (°Bx)	2.14	2.43	2.11	2.27	0.0010 ***	0.0448 *	0.1241
Non-soluble dry matter (%)	0.093	0.143	0.136	0.146	0.0010 ***	0.0026 **	0.0046 **
pH	5.45	5.60	5.19	5.21	0.1909	0.001 ***	0.3238
Titratable acidity (H^+^ mmol L^−1^)	7.71	6.09	13.94	14.21	0.5065	0.001 ***	0.3624
Fructose (%)	0.5525	0.5246	0.4081	0.4433	0.8879	0.0019 **	0.2416
Glucose (%)	0.4162	0.4291	0.3124	0.3447	0.4730	0.0140 *	0.7553
Sucrose (%)	n.d. ^y^	n.d.	n.d	n.d.			
Arabinose (%)	0.1060	0.1031	0.0822	0.0879	0.7569	0.0022 **	0.3625
Galactose (%)	0.0161	0.0193	0.0132	0.0137	0.2527	0.0202 *	0.3708
Xylose (%)	0.0005	0.0007	0.0004	0.0004	0.1382	0.0152 *	0.0827
Mannose (%)	n.d.	n.d	n.d.	n.d			
Rhamnose (%)	n.d.	n.d	n.d.	n.d			
Malic acid (%)	0.1297	0.1023	0.0836	0.0702	0.1327	0.0125 *	0.5850
Citric acid (%)	0.0056	0.0124	0.0643	0.0639	0.4688	0.0001 ***	0.4210
Succinic acid (%)	n.d.	n.d	n.d.	n.d			
Tartronic acid (%)	0.0017	0.0017	0.0016	0.0016	0.6643	0.0690	0.9398
Galacturonic acid (%)	n.d.	n.d	n.d.	n.d			

^z^ *, **, *** represent significant levels at 0.05, 0.01 and 0.001, respectively. ^y^ Not detectable.

**Table 4:** Effect of soil treatment and storage time on vitamins, amino acids and nucleosides of winter melon juice samples.

Storage Time (d)	Concentration (mg L^−1^)				ANOVA (Prob > |t|)		
	1		120		Soil Treatment (S)	Storage Time (T)	S × T
Soil Treatment	ASD	Control	ASD	Control			
Thiamine (Vitamin B1)	0.113	0.066	0.141	0.121	0.0690	0.0287 * ^z^	0.4166
Riboflavin (Vitamin B2)	0.031	0.031	0.019	0.017	0.7735	0.0005 ***	0.7573
Niacin (Vitamin B3)	0.022	0.032	0.011	0.010	0.3773	0.0063 **	0.2311
Pantothenic acid (Vitamin B5)	0.682	0.510	0.695	0.604	0.0155 *	0.2437	0.3746
Pyridoxine (Vitamin B6)	0.012	0.009	0.052	0.052	0.8732	0.0020 **	0.8610
Biotin (Vitamin B7)	n.d. ^y^	n.d.	n.d.	n.d.			
Cobalamin (Vitamin B12)	n.d.	n.d.	n.d.	n.d.			
Ascorbic acid (Vitamin C)	139.988	135.279	104.342	101.150	0.4560	0.0001 ***	0.8841
Total vitamins	140.848	135.928	105.260	101.955	0.0798	0.0001 ***	0.6582
Alanine	0.667	0.653	0.757	0.737	0.6947	0.0733	0.9371
Arginine	59.121	91.057	122.451	113.350	0.5127	0.0332 *	0.2533
Aspartate	21.612	15.943	76.286	60.589	0.1267	0.0001 ***	0.4468
Cysteine	n.d.	n.d.	n.d.	n.d.			
Glutamate	0.557	0.582	2.179	2.158	0.2843	0.0001 ***	0.3952
Glutamine	279.663	406.499	397.641	415.866	0.2718	0.3305	0.4027
Glycine	4.356	6.104	5.044	5.453	0.1770	0.9802	0.3849
Isoleucine	22.341	27.581	33.444	33.015	0.4255	0.0203 *	0.3514
Leucine	22.126	28.325	17.219	18.030	0.0942	0.0034 **	0.1828
Lysine	12.824	13.489	16.022	16.675	0.6045	0.0311 *	0.9964
Methionine	3.722	5.060	4.689	5.607	0.0632	0.1859	0.6983
Phenylalanine	27.227	33.834	46.009	48.373	0.3701	0.0078 **	0.6653
Proline	3.142	4.750	4.053	3.815	0.1108	0.9742	0.0422 *
Serine	10.367	9.597	10.685	11.062	0.7763	0.2176	0.4143
Threonine	6.981	8.930	9.710	9.941	0.3525	0.1289	0.4587
Tryptophan	75.083	75.835	77.331	77.424	0.9668	0.8504	0.9741
Tyrosine	34.093	41.208	48.777	48.932	0.2902	0.0082 **	0.3098
Valine	20.936	25.544	27.458	28.440	0.4517	0.2194	0.6217
Total amino acids	604.817	794.990	899.755	899.468	0.2591	0.0062 **	0.3564
Adenosine	29.619	26.295	14.651	13.459	0.1360	0.0001 ***	0.4565
Cytidine	1.671	0.700	1.558	0.940	0.0104 *	0.7972	0.4801
Guanosine	8.167	4.249	4.263	2.955	0.0743	0.0755	0.3353
Inosine	0.014	0.015	0.003	0.002	0.9147	0.0001 ***	0.5193
5-Methyluridine	n.d.	n.d.	n.d.	n.d.			
Uridine	2.319	2.432	1.167	0.834	0.7391	0.0027 **	0.5064
Total nucleosides	41.790	33.691	21.641	18.189	0.0262 *	0.0001 ***	0.2628

^z^ *, **, *** represent significant levels at 0.05, 0.01 and 0.001, respectively. ^y^ Not detectable.

**Table 5:** Effect of soil treatment and storage time on volatile profile of winter melon juice.

Storage Time (Days)	Concentration (ng mL^-1^)				ANOVA (Prob > |t|)		
	1		120		Soil Treatment (S)	Storage Time (T)	S × T
Soil Treatment	ASD	Control	ASD	Control			
Acetaldehyde	1.38	3.33	1.40	3.97	0.0176 * ^z^	0.7099	0.7322
Methanethiol	1.63	1.65	1.42	1.51	0.8270	0.4611	0.8703
Dimethyl sulfide	0.93	1.50	0.95	0.53	0.8121	0.1443	0.1266
Pentanal	2.04	2.49	2.03	2.44	0.0160 *	0.8678	0.9045
Dimethyl disulfide	1.21	1.70	1.12	1.46	0.0097 **	0.2641	0.6028
(*Z*)-3-Hexenal	4.34	6.32	4.31	6.22	0.0010 ***	0.9059	0.9391
Hexanal	299.26	340.17	293.92	340.42	0.0075 **	0.8643	0.8512
(*E*)-2-Hexenal	190.65	242.69	190.57	242.93	0.0004 ***	0.9946	0.9897
1-Hexanol	74.30	179.65	74.72	198.77	0.0265 *	0.8405	0.8472
Methoxy-phenyl oxime	6.13	5.31	6.18	5.09	0.2104	0.9081	0.8548
(*E*,*E*)-2,4-Hexadienal	4.06	5.39	3.95	5.52	0.0203 *	0.9908	0.8339
D-Limonene	8.58	1.70	8.71	1.69	0.0100 **	0.9806	0.9797
Nonanal	1.58	4.07	1.60	4.11	<0.0001 ***	0.9479	0.9917
(*E*,*Z*)-2,6-Nonadienal	13.98	22.37	14.01	22.44	0.0907	0.9917	0.9970
(*E*)-2-Nonenal	3.59	7.76	3.59	7.81	<0.0001 ***	0.9737	0.9742
Total	613.66	826.11	608.49	844.91	0.0002 ***	0.9532	0.9100

^z^ *, **, *** represent significant levels at 0.05, 0.01, and 0.001, respectively.

## Data Availability

The datasets generated in this study are available on request to the corresponding author.

## Supplementary Materials

The following supporting information can be downloaded at: https://www.mdpi.com/article/10.3390/foods12010209/s1, Table S1: LC-MS/MS MRM transitions and compound specific parameters.
