# Supplementary material for: Soil Amendment and Storage Effect the Quality of Winter Melons (Benincasa hispida (Thunb) Cogn.) and Their Juice
Source: Foods. 2023 Jan 3;12(1):209. doi: 10.3390/foods12010209 (PMC9818827; doi:10.3390/foods12010209)
Supplement: Supplementary file 1 [file foods-12-00209-s001.zip › foods-2073062-supplementary.pdf]

Table S1: LC-MS/MS MRM transitions and compound specific parameters

Compound Name	Precursor Ion	Product Ion	Fragmentor (V)	Collision Energy (V)	Ret Time (min)	Polarity
$^{13}C_6$-Fructose (Internal std)	**185.1**[z]	**61.2**	**69**	**10**	**4.8**	**Negative**
	185.1	92.1	69	10	4.8	Negative
5-Methyluridine	**259.1**	**127**	**166**	**5**	**3.9**	**Positive**
	259.1	110	166	37	3.9	Positive
Adenosine	**268.1**	**136.1**	**100**	**17**	**2.2**	**Positive**
	268.1	119.1	100	57	2.2	Positive
Arabinose	**149**	**59.2**	**66**	**12**	**4.9**	**Negative**
	149	89.1	66	4	4.9	Negative
Citric acid	**191**	**111**	**81**	**10**	**13.2**	**Negative**
	191	87	81	14	13.2	Negative
Cytidine	**244.1**	**112.1**	**140**	**13**	**4.4**	**Positive**
	244.1	95.1	140	53	4.4	Positive
Fructose	**179.1**	**88.9**	**69**	**5**	**4.8**	**Negative**
	179.1	59	69	13	4.8	Negative
Galactose	**179.1**	**89.1**	**70**	**10**	**6.9**	**Negative**
	179.1	59.2	70	14	6.9	Negative
Galacturonic acid	**193**	**59.1**	**166**	**17**	**11.4**	**Negative**
	193	113	166	9	11.4	Negative
Glucose	**179.1**	**89.1**	**69**	**5**	**6.4**	**Negative**
	179.1	59.1	69	9	6.4	Negative
Guanosine	**284.1**	**152**	**80**	**13**	**4.9**	**Positive**
	284.1	135	80	49	4.9	Positive
Inosine	**267.1**	**135**	**144**	**22**	**3.5**	**Negative**
	267.1	108	144	44	3.5	Negative
L-Alanine	**90.1**	**44.2**	**40**	**13**	**9.2**	**Positive**
	90.1	29.3	40	57	9.2	Positive
L-Arginine	**175.1**	**70.1**	**100**	**24**	**14.5**	**Positive**
	175.1	60.1	100	12	14.5	Positive
L-Aspartic acid	**132**	**88.1**	**70**	**9**	**11.6**	**Negative**
	132	115	70	9	11.6	Negative
L-Cysteine	**122**	**76**	**65**	**13**	**9.3**	**Positive**
	122	59.1	65	29	9.3	Positive
L-Glutamic acid	**146**	**102.1**	**80**	**13**	**11.6**	**Negative**
	146	128.1	80	9	11.6	Negative
L-Glutamine	**147.1**	**84.1**	**80**	**17**	**10.2**	**Positive**
	147.1	130.1	80	9	10.2	Positive
L-Glycine	**76**	**30.1**	**35**	**9**	**10.0**	**Positive**
	76	28.1	35	53	10.0	Positive
L-Isoleucine	**132.1**	**86.1**	**75**	**25**	**6.2**	**Positive**
	132.1	44.2	75	25	6.2	Positive
L-Leucine	**132.1**	**86.1**	**75**	**9**	**5.9**	**Positive**
	132.1	30.3	75	17	5.9	Positive
L-Lysine	**147.1**	**84.1**	**75**	**17**	**14.8**	**Positive**
	147.1	130.1	75	9	14.8	Positive
L-Methionine	**150.1**	**56.2**	**80**	**17**	**6.6**	**Positive**
	150.1	104.1	80	9	6.6	Positive
L-Phenylalanine	**164.1**	**147**	**80**	**9**	**5.6**	**Negative**
	164.1	103	80	15	5.6	Negative
L-Proline	**116.1**	**70.1**	**75**	**37**	**7.6**	**Positive**
	116.1	43.2	75	37	7.6	Positive
L-Serine	**106.1**	**42.2**	**67**	**24**	**10.2**	**Positive**
	106.1	88.1	67	8	10.2	Positive
L-Threonine	**120.1**	**74.2**	**80**	**9**	**9.6**	**Positive**
	120.1	56.2	80	17	9.6	Positive
L-Tryptophan	**205.1**	**146**	**80**	**20**	**6.2**	**Positive**
	205.1	188	80	8	6.2	Positive
L-Tyrosine	**182.1**	**91.1**	**85**	**33**	**7.3**	**Positive**
	182.1	136.1	85	33	7.3	Positive
L-Valine	**118.1**	**72.2**	**60**	**9**	**7.3**	**Positive**
	118.1	55.2	60	25	7.3	Positive
Malic acid	**133**	**115**	**76**	**8**	**11.9**	**Negative**
	133	71.1	76	14	11.9	Negative

Mannose	**179.1**	**59.2**	**66**	**16**	**5.4**	**Negative**
	179.1	89	66	4	5.4	Negative
Rhamnose	**163.1**	**103**	**166**	**5**	**2.7**	**Negative**
	163.1	119	166	5	2.7	Negative
Succinic acid	**117**	**99**	**166**	**9**	**12.3**	**Negative**
	117	73.1	166	9	12.3	Negative
Sucrose	**341.1**	**89.1**	**140**	**21**	**9.2**	**Negative**
	341.1	179	140	13	9.2	Negative
Tartronic Acid	**119**	**57.1**	**65**	**10**	**11.8**	**Negative**
	119	75.1	65	10	11.8	Negative
Uridine	**243.1**	**200.1**	**100**	**9**	**2.1**	**Negative**
	243.1	110.1	100	17	2.1	Negative
Vitamin B1	**265.1**	**122.1**	**90**	**13**	**7.8**	**Positive**
	265.1	81.1	90	37	7.8	Positive
Vitamin B2	**377.2**	**243**	**150**	**21**	**2.6**	**Positive**
	377.2	172.1	150	41	2.6	Positive
Vitamin B3	**124**	**78.1**	**135**	**25**	**4.9**	**Positive**
	124	53.2	135	37	4.9	Positive
Vitamin B5	**220.1**	**90.1**	**84**	**10**	**6.6**	**Positive**
	220.1	72.1	84	18	6.6	Positive
Vitamin B6	**170.1**	**152**	**100**	**13**	**1.8**	**Positive**
	170.1	134	100	21	1.8	Positive
Vitamin B7	**245.1**	**227**	**100**	**13**	**6.6**	**Positive**
	245.1	97	100	29	6.6	Positive
Vitamin B12	**678.3**	**359.1**	**165**	**26**	**11**	**Positive**
	678.3	635.8	165	22	11	Positive
Vitamin C	**177**	**95**	**65**	**9**	**6.8**	**Positive**
	177	141	65	5	6.8	Positive
Xylose	**149**	**71.1**	**69**	**5**	**4.0**	**Negative**
	149	59.1	69	13	4.0	Negative

[z]Transitions used for quantification are bolded.
